# Navigating Parenthood Alone: A Mixed-Method Study of Single Fathers’ Experiences and Needs Regarding Received Midwife and Child Health Nurse Support in Sweden

**DOI:** 10.1177/15579883251363107

**Published:** 2025-07-31

**Authors:** Wells M. B., Kerstis B.

**Affiliations:** 1 Women’s and Children’s Health, Karolinska Institutet, Solna, Sweden; 2 Division of Caring Sciences, School of Health, Care and Social Welfare, Mälardalen University, Västerås, Sweden

**Keywords:** single fathers, paternal mental health, coparenting, father–infant bonding, perinatal support, mixed methods research

## Abstract

This mixed methods study aimed to first quantitatively compare single fathers’ levels of depressive symptoms, coparenting relationship quality, and father–infant bonding with coupled fathers, and then explore single fathers’ experiences and needs regarding professional support during the perinatal and early parenting period. Fathers (*n* = 1,589, of which 25 were single fathers) completed an online survey regarding their depressive symptoms, coparenting relationship, and father–infant bonding. From the quantitative survey, six single fathers (mean age 35 years) consented to participate in an individual digital interview. Mann–Whitney *U* tests and chi-square tests were used to compare coupled and single fathers, while qualitative content analysis was used to analyze the interviews. Single fathers reported having more depressive symptoms and weaker coparenting relationships compared to coupled fathers but had similar levels of infant bonding. After qualitatively exploring single fathers’ professional support needs during the transition to parenthood, one overarching theme was emphasized: *Wanting to be an equal parent*. This theme broke down into three categories: *Desire to be included, Need of support* and *Relationship with the child.* Single fathers can benefit from professional clinical support, where this support helps foster stronger coparenting relationships, improves paternal mental health, and promotes gender equality in parenting. For this to happen, fathers in general, and single fathers specifically, need to be seen as clients, with their own care needs. Guidelines and recommendations should be reviewed and clarified to encourage more egalitarian parenting, including considering giving fathers their own medical records and individualized visits. There is a need for further studies regarding single fathers’ experiences and support needs.

## Introduction

Fatherhood is a key developmental milestone in adult life that can be fraught with diverse emotions, ranging from extreme joy to severe depression (Allport et al., 2018). Health professionals can assist fathers in this transition by providing psychosocial support (Wells & Aronson, 2021). Expectant fathers often personally benefit from, as well as benefit others, through their involvement during the prenatal period by supporting the mother and bonding with their baby (Plantin et al., 2011; Wells, 2016; Wells et al., 2022b). Early father involvement is positively associated with children’s emotional, cognitive, and social well-being (Allport et al., 2018; Plantin et al., 2011; Sarkadi et al., 2008). The World Health Organization (2015) and the American Academy of Pediatrics (Yogman et al., 2016) therefore recommend that reproductive and pediatric clinicians engage and involve fathers early in the infants’ life. After their child is born, fathers mentally, emotionally, and psychologically benefit from holding and caring for their infant, as well as learning about their child’s growth and development (Plantin et al., 2011; Wells, 2016). Having these experiences helps expectant and new fathers (hereafter referred to as ‘fathers’) bond with their baby; setting the scene for future father involvement in childrearing (Cabrera et al., 2008; Suzuki et al., 2022).

In high-income countries, including Sweden, the prevalence of single parenthood, where the father does not reside and/or does not have a romantic relationship with the mother, has increased over the past half century (Andersson et al., 2012; Lappegård et al., 2020). In Sweden, around one in four parents (25%) are not living with the other parent, 35% to 40% of children of separated parents live in shared custody (splitting about half their time with each parent), and out of all fathers, 7% of children live with single fathers (Bergström et al., 2019; Hallerbäck et al., 2025). While single fatherhood is often associated with increased challenges, particularly in terms of access to support and parenting resources, it does not inherently lead to poorer child outcomes—especially when parents maintain a cooperative, communicative, low-conflict, and non-violent relationship (Berman & Daneback, 2022). However, since little research has been completed from the fathers’ perspective, let alone single fathers’ perspective, especially during the infant time period, regarding their received and needed support from clinical professionals (Vidaurreta et al., 2022), further research is necessary on this population.

### Single Father Outcomes

Single fathers compared to cohabiting fathers report poorer health (Meadows, 2009; Westin & Westerling, 2006, 2007), as well as poorer lifestyle habits, increased health complications, psychological symptoms, hospitalizations, and higher mortality (Chiu et al., 2017; Rattay et al., 2017). In addition, single fathers are at increased risk of mental health problems, including mood disorders, such as depression, anxiety disorders, substance disorders (e.g., alcohol and illicit drugs), and suicide compared to cohabiting fathers (Cooper et al., 2008; Kong & Kim, 2015; Tobias et al., 2009). For example, as the fathers’ connections weaken, such as through couple separation or being excluded from caring for their child, suicide rates increase (Dehara et al., 2021). Single fathers are also more vulnerable because they have less social support compared to single mothers or coupled fathers (Chiu et al., 2017).

### Health Professionals’ Support

Reproductive and pediatric clinicians support parents as part of their routine job duties, making them helpful allies in providing universal care (Fisher et al., 2018). Psychosocial supports can benefit fathers by helping them adjust to their parental role (Vallin et al., 2019; Wells, 2016; Xue et al., 2018), and develop coparenting skills (Wells et al., 2022a), as well as reduce depressive symptoms (Wells & Aronson, 2021). In addition, professionals can aid fathers in bonding with their infant, such as promoting them to hear and see their infant during the prenatal period, such as through heart rate monitors and ultrasounds (Plantin et al., 2011; Wells, 2016; Wells et al., 2022b), as well as encourage them to participate in the birth of their child and find ways for physical contact between father and child, including cutting the umbilical cord, having skin-to-skin contact, performing infant massage, and aiding in breastfeeding (Scism & Cobb, 2017). In fact, the more active involvement fathers have in antenatal care, the more trust mothers may place with fathers, and involve him more (Scism & Cobb, 2017).

However, fathers who are excluded by reproductive and pediatric professionals often lack knowledge and have parental adjustment difficulties, which can lead to less involvement in childrearing (Allport et al., 2018; Baldwin et al., 2018; Baysal et al., 2022; Wells, 2016; Werner-Bierwisch et al., 2018; Xue et al., 2018). Single fathers may be at greater risk of lacking professional support than coupled fathers if the mother does not permit them to join during prenatal and postnatal visits and/or at the labor/birth ward.

Reproductive and pediatric care is free-of-charge in Sweden. Swedish midwifery policies (Lundgren & Berg, 2016; Region Stockholm, 2022) and child health policies (Rikshandboken Barnhälsovård, 2018) state that all parents, respectively, should be confirmed in their parental role and be offered psychosocial support to promote responsible parenting. During normal pregnancies, parents receive 9 to 11 prenatal visits, and can participate in 4 to 5 parent group classes (SFOG, 2016). During the infants’ first year, there are at least nine scheduled visits with a child health center (CHC) nurse, including three visits where fathers should be explicitly invited (Rikshandboken Barnhälsovård, 2020) and 8 to 10 parent group classes during the infant’s first year (Elfström et al., 2018).

Despite the availability of universal and free perinatal and child health services in Sweden, many fathers can feel unseen, unheard, and unsupported from prenatal care, childbirth, and child health care in Sweden (Wells, 2016). In fact, expectant and new fathers face a societal dilemma, where they are expected to fully participate in childrearing (Lundqvist, 2011; Tan, 2023), and yet, receive no formal clinical support that is attached to their own medical record during the prenatal-postnatal period, as they are neither patient nor visitor (Steen et al., 2012). One group of fathers that might be particularly vulnerable are single fathers, who remain an under-researched and potentially underserved group, in part because they often lack the social and clinical support structures available to coupled fathers and face greater risks of psychological distress (Hallerbäck et al., 2025). Existing studies have largely focused on mothers or coupled fathers, leaving a gap in understanding how single fathers experience and access support during the critical transition to parenthood. To address this gap, the current study employed a sequential explanatory mixed methods design to first quantitatively compare single and coupled fathers on depressive symptoms, coparenting relationship quality, and father–infant bonding, and then qualitatively explore single fathers’ lived experiences and perceived support needs during the perinatal and early parenting period.

## Method

### Study Design

This study employed a sequential explanatory mixed methods design (Creswell & Creswell, 2017), guided by a structured, post-positivist epistemological stance to gain a deeper understanding of single fathers’ needs of support during parenthood. This perspective assumes that while reality can be systematically studied, it is always interpreted through the lens of the researcher. The quantitative phase aimed to describe and compare single and coupled fathers on depressive symptoms, coparenting relationship quality, and father–infant bonding using validated instruments. This informed the researchers that single fathers were an at-risk group that might need greater levels of clinical support as they transition into parenthood. The subsequent qualitative phase was designed to expand upon and contextualize these findings by exploring single fathers’ lived experiences and perceived support needs—dimensions not fully captured by the standardized measures. This integration allowed for a more comprehensive understanding of single fathers’ adjustment to parenthood and their interactions with healthcare systems. The mixed methods approach was chosen to triangulate findings, enhance validity, and generate practical recommendations for improving father-inclusive care (Dures et al., 2011).

### Participant Recruitment

Single fathers from Sweden were recruited from a large national cohort study in 2022 called the Pappor/Icke-Födande Föräldrar study (PIFF; Fathers/Non-birthing Parents Study). To participate, participants needed to: (a) identify themselves as single fathers (e.g., not living together with the child’s mother), (b) of infants (0–24 months old), (c) living in Sweden. Of the 2,427 fathers who completed the larger survey, 40 identified as single fathers (1.6%). When completing the survey, participants could: (a) submit their survey halfway through or continue to complete three validated scales relating to depressive symptoms, the coparenting relationship, and father–infant bonding and (b) choose to remain anonymous or provide an email address to participate in future studies. Of the 40 single fathers, 25 completed the three validated scales, and 26 provided an email address to potentially participate in a qualitative study. Around 9 months after completing the initial survey, the 26 single fathers who provided an email address were contacted to participate in a qualitative interview study. Six consented to participate, while 20 did not respond.

### Quantitative Data Collection

The Edinburgh Postnatal Depression Scale (EPDS) was used to detect fathers’ depressive symptoms. The EPDS is a self-report scale consisting of 10 items, each on a 4-point Likert scale, with response options ranging from 0 = *never* to 3 = *most of the time* (Cox et al., 1987). On the EPDS, higher scores indicated greater severity of depressive symptoms, where a cut-off ranging from 7 to 10 indicated having depressive symptoms (Shafian et al., 2022). In the current analysis, we used ≥10 points to detect depressive symptoms. Cronbach’s alpha in the current study was .87.

The Brief Coparenting Relationship Scale (B-CRS) was used to assess the coparenting relationship. The B-CRS takes 2 items from each of the seven subscales on the Coparenting Relationship Scale (35 items): coparenting agreement, coparenting closeness, exposure of child to conflict, coparenting support, coparenting undermining, endorsement of the partners’ parenting, and division of labor. The two items from the division of labor, however, did not load well, and thus, the Swedish version of the B-CRS, which has been validated with fathers, is recommended using 12 items (Lee et al., 2021). Each item is on a 7-point Likert scale ranging from 0 = *not true of us* to 6 = *very true of us*, where higher scores indicate more positive coparenting. The Cronbach’s alpha in the current study was .84.

The Parent-Infant Bonding Questionnaire (PBQ) was used to assess bonding disturbances (Brockington et al., 2001). The PBQ is comprised of 25 items, resulting in four subscales: impaired bonding (12 items), rejection and anger (7 items), anxiety about care (4 items), and incipient abuse (2 items). The fourth subscale was not used during data collection. Each item is on a 6-point Likert scale ranging from 0 = *always* to 5 = *never*, where higher scores indicated bonding disturbances. The PBQ has been validated (Brockington et al., 2006) and well-used with fathers in Sweden (Edhborg et al., 2005; Kerstis et al., 2016; Wells & Jeon, 2023). The items were summed to create a total PBQ score. Cronbach’s alpha in the current study was .97.

### Qualitative Data Collection

A semi-structured interview guide (see Supplemental Material) was created and emailed to participants along with an information letter and consent form. The interview guide consisted of questions pertaining to the fathers’ experiences of received and needed support within prenatal care, during childbirth, and at the CHCs. All interviews were conducted via Zoom with a female midwife (A.N.) who had experience in conducting qualitative interviews. Participants chose to have their camera on or off and were advised that only the audio recording would be saved from the interview for later transcription. During the interview, only the participant and the interviewer were present. During the interviews, participants were asked to elaborate, give examples, and clarify when answering questions. The interviews ended by asking if the participants wanted to discuss anything else. Interviews ranged between 42 min and 1 hr 16 min, with an average length of 1 hr.

### Data Analysis

#### Quantitative Analyses

Descriptive statistics were used to analyze the quantitative data, including frequencies and percentages for categorical variables, and means with standard deviations for continuous variables. The chi-square test examined the relationship between fathers living together with the mother (coupled vs. single) and depressive symptoms (yes/no), while the Mann-Whitney *U* test compared fathers living together with the mother and the coparenting relationship and father–infant bonding, respectively. All tests were two-tailed with significance at *p* ≤ .05, using IBM SPSS Statistics (v. 29.0; IBM Corp., Armonk, NY, USA).

#### Qualitative Analyses

Four researchers helped analyze the qualitative data. The qualitative content analysis (QCA) was conducted in line with Graneheim and Lundman (2004) framework. This approach is consistent with our post-positivist stance, as it allows for systematic analysis of textual data while acknowledging the interpretive role of the researcher. The method’s theoretical grounding in meaning-making and categorization supports the exploration of participants’ lived experiences and aligns with the study’s aim to contextualize quantitative findings. To ensure trustworthiness, we followed Graneheim and Lundman (2004) criteria, including prolonged engagement with the data, investigator triangulation, and iterative discussions among the research team.

The interviews were therefore analyzed following the steps for QCA inspired by Graneheim and Lundman (2004) to contribute to the understanding of single fathers’ support needs and experiences. The interviews were transcribed verbatim by (removed for peer review) and then, in the first step, re-read and re-listened to check for the accuracy of the transcription. Two novice midwifery researchers (R.D. & Y.L) took the lead in initial coding of the data, with a third experienced public health qualitative researcher providing oversight and guidance (M.B.W.; all male). The texts were read several times to give the researchers a thorough understanding of its content. Secondly, meaning units, such as words, sentences, or sections related to the study aim, were identified in the text (Table 1). With the analysis, meaning-bearing units were identified, which were then compressed and summarized into codes, and after comparing similarities and differences, the results yield one main theme comprised of three categories and six sub-categories (Table 2). Although QCA allows for the quantification of codes or categories, we did not pursue this approach, as our goal was to explore the depth and nuance of participants’ experiences rather than measure frequency. Given the small sample size (*n* = 6), quantification was not deemed appropriate, as it could imply generalizability beyond what the data support. However, credibility was enhanced through multiple readings, coding by multiple researchers, and consensus-building on themes. A fourth researcher (a female pediatric nurse; B.K.) and experienced qualitative analyst joined the project after the themes and categories were created. Together with the third researcher, B.K. re-read the qualitative content and further developed the final themes, categories, and sub-categories, agreeing on the final version, and adding additional credibility to the findings.

**Table 1.** Example of the Process of Analysis.

Meaning unit (quote)	Condensed meaning units	Code	Sub-categories	Categories
But, but the birth itself, in general, I still think … I was so grateful that I was included.	Grateful to be included	To be included	To interact with the mother	Desire to be included
To achieve the child’s best interests, the parents must be well. But often the focus is only on the mother’s well-being.	The professionals need to see both parents	The desire to be seen	To need professional support	Need of support
You kind of see that he’s got a connection to me now, so our connection has happened now, but I didn’t feel it from the start.	He has a connection with me now, our connection, was not felt from the start	More time spent with the child positively affects bonding	To bond with the child	Relationship with the child

**Table 2.** Overview of the Subcategories, Categories, and Main Theme.

Sub-categories	Categories	Theme
To interact with healthcare professionals To interact with the mother	Desire to be included	Wanting to be an equal parent
To need professional support To need social support	Need of support	
To bond with the child To maintain the connection with the child	Relationship with the child	

This study used a structured, post-positivist perspective, recognizing the complexities and limitations in fully understanding the experiences of single fathers. Our use of structured methodologies, including surveys and statistical analyses, facilitated triangulation of the findings, thereby enhancing the validity of our understanding of the support needs of single fathers. By systematically analyzing both quantitative and qualitative data, we were able to provide practical recommendations for improving prenatal and postnatal care for single fathers in modern Sweden. This approach ensures that our findings are robust, contextually sensitive, and actionable.

### Ethics Statements

An information letter and consent form were both emailed to the participants and re-read verbally before the online interviews via Zoom took place. The informed consent to participate was verbal. Participants were assured that their participation was voluntary, and that withdrawing was possible whenever they wanted. This current study received ethical approval from Stockholm’s Regional Ethics Board (dnr: 2018/889-31/5).

## Results

In considering the quantitative results, 1,589 fathers completed the three validated scales, where 25 were single fathers (Table 3). When comparing coupled and single fathers, single fathers had significantly more depressive symptoms: χ^2^(df = 1, *N* = 1,595) = 4.4, *p* = .036, where 40% of single fathers reported ≥10 points on the EPDS compared to 22.3% of coupled fathers. Similarly, single fathers (*M* = 64.2, *SD* = 17.3) had significantly lower B-CRS scores compared to coupled fathers (*M* = 84.7, *SD* = 9.6), *U* = 6210.00, *z* = −5.87, *p* < .001. However, father–infant bonding was not significantly different between single (*M* = 12.8, *SD* = 8.5) and coupled fathers (*M* = 16.8, *SD* = 19.0), *U* = 17454.50, *z* = −0.89, *p* = .375.

**Table 3.** Coupled and Single Fathers’ Depressive Symptoms (Chi-Square Test), As Well As the Coparenting Relationship and Father–Infant Bonding (Mann-Whitney *U* Test, Respectively).

	Coupled fathers (*n* = 1,557–1,570)	Single fathers (*n* = 25)	*p*-value
	*N* (%)	*N* (%)	
Depressive symptoms (≥10)			
Yes	350 (22.3)	10 (40)	.036
No	1,220 (77.7)	15 (60)	
	*M* (*SD*)	*M* (*SD*)	
Coparenting relationship	84.7 (9.6)	64.2 (17.3)	<.001
Father–infant bonding	16.8 (19.0)	12.8 (8.5)	.38

*Note. SD* = standard deviation.

Regarding the qualitative results, five had been in a romantic relationship with the mother of the child, which ended during or after the pregnancy. One man met a woman via a forum for people looking for a co-parent to conceive a child; therefore, they had no prior relationship and only met with the aim of producing a child together. Three of the fathers had joint custody, while one father had sole custody and one mother had sole custody of their child, respectively. The infants were between 11 and 21 months, with an average age of 16 months. Five of the fathers had children for the first time and one had a child before. The fathers were between 28 and 43 years old, with an average age of 35 years. Half of the fathers (*n* = 3) had planned the pregnancy. One child was born prematurely (before week 37). One main theme, *Wanting to be an equal parent*, was identified from the qualitative analysis. Three categories *Desire to be included, Need of support* and *Relationship with the child* constituted this main theme.

### Wanting to Be an Equal Paren

This main theme helps to highlight single fathers’ deep-seated desire to be recognized, included, and supported as legitimate and capable caregivers throughout the perinatal and early parenting period. Fathers in this study consistently expressed a yearning to be seen not merely as secondary figures or adjuncts to the mother, but as autonomous parents with their own emotional needs, caregiving capacities, and rights to participate in their child’s development. Participants described feeling marginalized by healthcare systems that often failed to proactively include them in prenatal visits, childbirth, and child health services. Their narratives reveal how exclusion from these critical touchpoints hindered their ability to bond with their child, as well as also contributed to feelings of invisibility, emotional distress, and diminished parental confidence. At its core, this theme underscores the importance of equitable, father-inclusive care models that affirm single fathers’ roles and foster their engagement as full and equal partners in parenting.

### Desire to Be Included

Single fathers wanted to be included and that professionals should see them, be available, and ask questions about their life situation. They wanted to talk undisturbed and receive answers to their questions, to have a sense of security and be more bonded to their child. One father said: *They asked me how I was and how it felt. Funny that they asked me…there I was given the opportunity to ask questions… they took me away for a reason to include me … that the midwife really saw me there.*

As the prenatal visits were only booked through and for the mothers, fathers felt they had no voice or control. Fathers reported that the mothers decided whether they could join in the visits or not, which they said limited their ability to prepare for and adjust to their new parental role. Fathers further stated that the CHC visits were also booked and planned according to the mothers’ wishes, even though these visits should focus on the child’s health, and thus both parents should be able to attend. This resulted, the fathers stated, in mothers holding additional power via information. Since the healthcare professionals primarily spoke with the mother, fathers reported that they often had to hear about their child’s health and development from the mother and that the mother could deny them permission to join visits. Fathers described that if the mothers involved and encouraged them to join visits and to actively participate, they felt important and like a real parent.

Fathers stated that the healthcare system lacked an inviting way to involve those who were not in a romantic relationship with the child’s mother. Although they emphasized that they had the will to be an involved parent, the exclusion from the healthcare system was stated as a perceived barrier to learning more about their child and how to parent in an evidence-based way. One father stated: *It was incredibly hard not to be heard and not to be needed. I felt ostracized.*

Fathers described that they had to make more effort than mothers to be involved in their child’s life. They reported that they faced resistance from the child’s mother, societal norms, and healthcare professionals. Although fathers sometimes felt unfairly sidelined, they expressed the idea of having to accept their situation, noting that they were more of a background figure rather than not involved at all.

When fathers were invited and allowed to participate, they were overjoyed and connected to their child. The ultrasound was described as important by all fathers, as they could see their child for the first time. Fathers also expressed joy and meaningfulness in attending the birth of their child. However, fathers experienced uncertainty in how they could contribute after the baby was born and appreciated when the midwife spoke to them and gave advice. One father, while positively reflecting on his experiences stated: *The midwife saw me and included me … she wanted to listen to my experiences.*

### Need of Support

Since fathers did not physically carry their child during pregnancy, they stated that their transition to parenthood was something new and unknown and therefore wished that the health professionals supported them in their parental role and guided them through their child’s growth and development. When fathers did attend healthcare visits, they were not necessarily supported as an equal parent. For example, fathers reported that they were sometimes overlooked and neglected by healthcare professionals, as they only made eye contact with and directed all questions toward the mother. Being present and listened to at the prenatal and/or child health visits decreased their concerns, worries, and uncertainty and strengthened their feelings of support.

Fathers sometimes felt less important and valuable in their role as a father and in their coparenting relationship when the focus was primarily on the mother and child. They described how health professionals only contacted the mother for the different visits. This made them feel inferior and highlighted that healthcare professionals did not see their value to their child’s growth and development. To compensate, fathers reported that they instead had to directly contact the healthcare professionals to get information about their child. They experienced that actively seeking information increased their exclusion from their child and discouraged involvement, as it emphasized that they were not approached by healthcare professionals, making them unworthy of receiving parenting information and support.

Fathers often reported that they were not supported. When fathers were excluded from care, they reported that their mental health deteriorated. They therefore expressed a need to be asked, and wanted to talk about, their current mental health status, including feeling scared, fearful, lonely, stressed, anxious, and depressed. As one father described: “I would have liked to be able to tell you about my condition and my concerns. I might have gotten professional help on how to handle the situation.” They also expressed having questions they wanted to ask privately but were sometimes hindered if the mother was present. One father said: *The whole situation* [being separated] *from the mother made me very sad and depressed. But since I couldn’t talk to the midwife privately at the antenatal care, I let it be.*

As a resolution to this, fathers requested to be treated as equal parents who receive individualized care. In other words, to be treated like a “patient/client” with their own medical record. By having their own medical record, fathers believed they could be routinely included in their child’s care and that their own care needs would be discussed and documented and thereby seen as a parent with their own needs of support. As a part of this, fathers desired having individual father visits during the prenatal and CHC visits, respectively, and thought that this could lead to creating a stronger relationship with their midwife and CHC nurse, in addition to receiving more support that could help them transition into their parental role. These individualized visits were requested as fathers wanted a space where they could talk about their own health and well-being, as well as adjusting to their new life situation, including becoming and being a father, as well as their specific situation of being a single father of an infant.

Under the current system, however, where fathers discussed how they were limited in receiving professional support, they reported that they instead turned to social support including father groups and forums on social media to increase their social parenting networks. New friendships via these social support mechanisms helped the fathers get in touch with others in similar situations. This support was helpful toward understanding their lives and routines and helping them adjust to parenthood. Fathers also felt that societal norms affected their parenting, which they thought contributed to mothers being seen as the primary parent through the traditional image of mothers taking care of the infant and fathers taking care of everything around them. They wanted to be seen as an equal parent, but that societal norms often focused on their biological sex as men and thus limited supporting them in their parenting. One father concluded stating: “I hope that there can be a better view of fathers in society.”

### Relationship with the Child

Fathers stated they were at the mercy of the mothers’ wishes regarding whether they could attend prenatal care, childbirth, or the CHC visits. If they were not allowed to attend their child’s birth, they expressed sadness and emptiness which they said could negatively affect the father–infant bond. One father described: *It’s one of the biggest things in life and you can’t be part of it. I was sad because it was something huge that I didn’t get to be a part of.* Another father stated: *I wasn’t allowed to attend the maternity care at all … I wanted to be there for the birth of my child, but I wasn’t allowed to be there either … it was almost traumatic… terribly difficult.*

Fathers believed that their most important, and long-awaited, priority was their connection and relationship to their child. Fathers wanted to participate during pregnancy and throughout the child’s life. They wanted to start bonding with their baby as early as possible. When fathers were not given the opportunity to participate in the prenatal visits, they expressed that this made it difficult to create a connection and relationship with their child. Since they could not feel their baby or, for example, hear their baby’s heartbeat during the prenatal visits, fathers stated that attending the ultrasound was a way to create a first relationship with their child and a realization that they were going to be a father. When the child was born, fathers described that it was important to be able to spend time with their child to develop a strong bond. Fathers believed that the father–infant bond was something that developed over time and for this to be possible, they needed to spend quality time via interacting with their child. Fathers requested to spend whole days with their child where there was time to play, eat together, and that the child slept over at their residence. Fathers perceived that doing this helped their child develop trust and security with them and with that, an attachment to them. All fathers described an improved infant bond and relationship when they were allowed to be close and a part of their child’s life.

Fathers stated that mothers sometimes prevented them from being with their child. For example, they stated that mothers used breastfeeding as a reason why the fathers could not have custody or spend time with their child. Fathers believed that while breastfeeding was important, their involvement with their child was also important. As such, they suggested different solutions such as supplementing breastfeeding or having time in-between breastfeeding for them to spend quality time with their child.

By directly participating in childrearing, fathers developed a parental identity, giving them a new meaning to life, and helped them develop and maintain a positive coparenting relationship. However, if mothers excluded the father from childrearing and attending health visits, then they found it difficult to create a relationship with their child. Due to the maternal power positions during the infant period, fathers stated that they were more like a helper to the mother than a parent playing an active role with their child. As such, the fathers did not have a well-working coparenting relationship and a distant relationship with their child.

Fathers reported that going through the court for child custody issues was a time-consuming and difficult period, where they did not have many opportunities to be close to their child. They perceived that the courts were more likely to provide mothers with custody, and that the bar was higher for them to gain custody of their child. As such, these fathers had a fear of losing contact with their child. They often shared their enthusiastic desire to be with their child and wanting to build a strong bond and relationship with their child. Once joint custody was awarded, it had an impactful positive mood change, as they were now allowed to spend more time with their child to develop a stable relationship. If the mother lived in another city, fathers went through custody disputes and had to commute long distances to see their child. This was emotionally and financially challenging but was something they felt they had to do, as they wanted to be involved in their child’s upbringing.

## Discussion

Single fathers reported having more depressive symptoms and weakened coparenting relationships compared to coupled fathers but had similar levels of infant bonding. After qualitatively exploring single fathers’ professional support needs during the transition to parenthood, one overarching theme was emphasized: *Wanting to be an equal parent*. This theme broke down into three categories: *Desire to be included, Need of support*, and *Relationship with the child.* Single fathers wanted to be included and desired professional clinical support from both midwives and CHC nurses in their transition to parenthood. They described that prenatal visit, childbirth, and CHCs were not always adapted to single fathers’ needs and that they sometimes felt neglected and unsupported.

### Fathers’ Need for Support and Involvement

Single fathers desired to be seen and supported as a client/patient with their own individual medical and mental health needs, similar to previous studies on other non-birthing parents (Klittmark et al., 2018; Wells, 2016; Wells & Lang, 2016). Poh et al. (2014) literature review reported that fathers want more support and information, and to be involved and respected by the healthcare staff. This is also described by Baldwin et al. (2018), where fathers wanted more guidance and support to prepare for future parenthood, but often lacked information and confirmation from healthcare professionals. Single fathers therefore found themselves in a gray area, where they are neither patients nor visitors (Steen et al., 2012), which made them feel unseen and not treated as an equal parent (Baldwin et al., 2018).

Clinical staff may therefore treat single fathers in a way that reinforces norms where they have less importance, commitment and skills in the direct and close care of their child (Hyllander & Jacobson, 2018). Single fathers may be even more excluded from care since coupled fathers are often invited to visits via their partner (e.g., the expectant or new mother) and have a healthier coparenting relationship. In contrast, single fathers in this study reported weaker co-parenting relationships, leading to poorer communication and less awareness of their child’s growth and development. Single fathers reported that they were often not invited to attend visits by healthcare professionals or were not permitted to attend by the child’s mother. Not receiving professional support from midwives and child health nurses is associated with greater levels of depressive symptoms in fathers (Wells & Aronson, 2021). Since single fathers reported greater levels of depressive symptoms compared to coupled fathers in the current study, and because paternal depression is associated with several negative child outcomes (Ashraf et al., 2023; Sweeney & MacBeth, 2016), finding ways to fully include, involve, and support single fathers from the prenatal through early childhood period could help them better adjust to their new parental role and thus mitigate depressive symptoms. Consequently, single fathers wanted their own medical record at prenatal and child health clinics so that they could receive individual support, and thus, better adjust to parenthood.

Single fathers were not always offered contact with health care. This may be because guidelines are not clear and leave the decision up to the responsible healthcare staff to contact him or, more likely, to contact only the (expectant) mother. According to Swedish guidelines, prenatal care and the CHCs should offer support to all parents, as well as offer enhanced support when needed (National Board of Health and Welfare, 2015). For example, CHCs should offer fathers/non-birthing parents an individual visit when the child is 3 to 5 months old to promote parental support and establish contact (Rikshandboken Barnhälsovård, 2019). To promote parental support, both parents should be contacted, but with current guidelines, the non-birther risks not being contacted. In Wells et al. (2017) study on nurses’ attitude and experience of supporting both parents, most nurses working at CHCs agreed that it is important to have a good relationship with both parents, but thought they had a greater competence for supporting mothers. Single fathers may be a unique group, where CHC nurses require additional support and training on how to best reach and support these single fathers. Future research is needed to better understand single fathers’ unique needs, as well as how health care systems can better involve and support them.

### Mental Health and Emotional Challenges

Single fathers described that they wanted to be asked about and receive support for their mental health in connection with their transition to parenthood. The CHC ‘s basic program includes inviting fathers/non-birth parents to an individual visit where, among other things, questions about mental health are included (Rikshandboken Barnhälsovård, 2019). However, the basic program and guidelines have no effect if single fathers are not invited to the individual visits. In the total sample of 40 single fathers, 65% were never invited by the nurse, and 48% did not attend the visit. This may be because (a) the care staff do not have contact details for single fathers as they are not a patient/client, (b) there may not be consistent guidelines on how to invite and involve single fathers, and/or (c) nurses may lack the overall interest or skill to support fathers in general, as less than one-third of CHC nurses reported that they had the same competence to support both mothers and fathers (Wells et al., 2017).

Single fathers wanted individualized care and to be able to ask questions without the mother present. As fathers benefit from receiving professional support (McNab et al., 2022), including decreasing their depressive symptoms (Wells & Aronson, 2021) and strengthening their coparenting relationship (Wells & Jeon, 2023), individualized visits from the prenatal period, up to when the child is 2 years old, can further benefit fathers by preparing and adapting to fatherhood. Single fathers expressed that they wanted clinical professionals to ask about their mental health. Davenport et al. (2022) describe that fathers do not tend to seek help for their depressive symptoms; therefore, clinical staff may need to initiate the dialogue, so fathers receive the support they need.

### Bonding and Bonding Obstacles

Single fathers had a strong desire to bond with their children, which helped them to be affirmed as fathers and adjust to their parenting role. Their relationship and bond with their child were seen as something essential to feeling like a father. Single fathers who were allowed to attend the clinical visits and birth stated that these interactions helped them better adjust to parental life, while those who were excluded, especially from the ultrasound visits and/or birth led to them missing opportunities to bond with their child. Being present and involved at birth is seen as a life-changing event for fathers (Dellmann, 2004; Johansson et al., 2015). Fathers’ adjustment during this transition is crucial for children’s emotional, social, cognitive, and academic development, as well as their overall well-being, as their better adjustment is linked to fewer behavioral problems, higher self-esteem, better cognitive outcomes, and greater academic achievement (Diniz et al., 2021; Islamiah et al., 2023). On the other hand, direct father involvement during birth becomes complicated if the mother does not want him there, as this impinges on her own autonomy. Direct father involvement during childbirth can create tension when the mother prefers to have him absent, as this decision directly affects her autonomy and comfort during a vulnerable moment. Such situations highlight the broader challenges of coparenting, where differing desires and boundaries can complicate the relationship and decision-making process. Future research and clinical care will need to work with how to best support these singles fathers, while maintaining female bodily autonomy.

### Coparenting Obstacles

Single fathers faced various challenges when trying to be included in their child’s life. They were felt alone in their parenting if they were not part of a coparenting relationship with the mother. Similar to previous research (Wells et al., 2017), the current participants described that the mother often was seen as the primary caregiver and that they were often sidelined regarding parenting and participating in their child’s health care. However, midwives and child health nurses can help promote and strengthen the coparenting relationship by seeing and supporting fathers via having them attend and participate at prenatal and postnatal visits (Wells et al., 2022a). Clinicians utilizing evidence-based practices that promote strong coparenting relationships are important, since stronger coparenting relationships predict several positive child outcomes, greater paternal involvement, and greater parental well-being (Campbell, 2023). Future clinical work and research should focus on ways to better promote the coparenting relationship of single or separated parents to find the best mechanisms that work well for this group, so that both parents receive the support they need to adjust to their new parental life.

### Methodological Considerations

This is the first study in Sweden, to our knowledge, on single fathers’ experiences and professional support needs during the prenatal and postnatal time periods. Nevertheless, the current study has several limitations. First, there is a relatively small number of single fathers in the quantitative data, as the vast majority of fathers having babies are coupled; thus, each individual single father can impact the findings. Although the Mann-Whitney *U* test is appropriate for comparing groups with unequal sizes, the small number of single fathers (*n* = 25) relative to coupled fathers (*n* = 1,564) limits the statistical power and generalizability of the findings. Results should therefore be interpreted with caution, and future studies with larger and more balanced samples of single fathers are needed to confirm these findings.

In addition, there were only six fathers who participated in the qualitative interviews. However, the interviews expand on the results of the quantitative analyses, where single fathers consistently reported mental ill-health and coparenting difficulties, while stressing the importance of infant bonding. Moreover, the interviews were of great depth, with an average of 1 hr and had consistently content-rich data, resulting in three categories. The trustworthiness of the results was ensured by thoroughly describing participants, data collection procedures, and the analytic steps, and that the naming of the categories was discussed and agreed among the co-authors. Credibility was strengthened through multiple readings of the transcripts, collaborative coding by researchers with different levels of qualitative experience, and consensus-building on the final themes. Dependability was supported by clearly documenting the analytic process, including how codes and categories were developed and refined over time. Transferability was supported by providing rich descriptions of the participants, context, and data collection procedures, allowing readers to assess the applicability of findings to other settings.

The authors have different backgrounds in pediatric nursing including CHC and public health, as well as differing levels of experience with interviewing and with research, which contributed to investigator triangulation and enhanced the credibility of the analyses. Furthermore, this study used a structured, post-positivist perspective, while recognizing the complexities and limitations in fully understanding the experiences of single fathers. Our use of structured methodologies, including surveys and statistical analyses, has helped in triangulating the findings, further enhancing the validity of understanding the support needs of single fathers (Dures et al., 2011). This approach has enabled us to provide practical recommendations for improving prenatal and postnatal care for single fathers in modern Sweden. There is a need to carry out further studies on single fathers’ experiences and support needs, in addition to finding best practices to support this at-risk group of parents.

## Conclusion

The transition to fatherhood as a single father can mean having to go through a tough and lonely phase without the support of a co-parent. Furthermore, they could benefit from professional clinical support to help them adjust into parenthood. When single fathers received professional support and had a strong coparenting relationship, then they reported improved adjustment, while those fathers who felt left out and not included within the healthcare system reported several negative psycho-social outcomes and poorer coparenting relationships. This sensitive period can mean that fathers are exposed to a higher risk of suffering from mental illness, which in turn can negatively affect the child. To promote the health of single fathers, they need support and early involvement in the child’s care. Guidelines and recommendations in prenatal care, delivery, and CHCs should be reviewed and clarified to encourage more egalitarian parenting where both the father and mother are seen as primary parents, to promote the health of all parents and children.

## Supplemental Material

sj-docx-1-jmh-10.1177_15579883251363107 – Supplemental material for Navigating Parenthood Alone: A Mixed-Method Study of Single Fathers’ Experiences and Needs Regarding Received Midwife and Child Health Nurse Support in Sweden Supplemental material, sj-docx-1-jmh-10.1177_15579883251363107 for Navigating Parenthood Alone: A Mixed-Method Study of Single Fathers’ Experiences and Needs Regarding Received Midwife and Child Health Nurse Support in Sweden by Wells M. B. and Kerstis B. in American Journal of Men's Health
