# Supplementary material for: Navigating Parenthood Alone: A Mixed-Method Study of Single Fathers’ Experiences and Needs Regarding Received Midwife and Child Health Nurse Support in Sweden
Source: Am J Mens Health. 2025 Jul 31;19(4):15579883251363107. doi: 10.1177/15579883251363107 (PMC12317203; doi:10.1177/15579883251363107)
Supplement: sj-docx-1-jmh-10.1177_15579883251363107 – Supplemental material for Navigating Parenthood Alone: A Mixed-Method Study of Single Fathers’ Experiences and Needs Regarding Received Midwife and Child Health Nurse Support in Sweden [file sj-docx-1-jmh-10.1177_15579883251363107.docx]

Interview Guide

1. How have you experienced parenthood so far?
2. Can you tell me about your experiences with antenatal care during the pregnancy period?
   1. What kind of support did you feel you needed?
   2. What support did you feel you received from the midwives during the pregnancy, childbirth, and postnatal care?
3. Did the COVID-19 restrictions affect you and your family? If so, how?
4. How could midwife-led care be changed to better support you and your family?
5. Are you aware that child health nurses (CHC) are supposed to invite fathers to specific visits at the child health center?
   1. Can you tell me what you know about those visits?
6. Can you describe your thoughts on child health nurses increasingly trying to include fathers at the child health center?
7. Can you describe your visits to the child health center (CHC):
   1. The home visit
   2. The 3–5 week visit (now the 1–3 week visit)
   3. The 3–5 month visit
   4. Have you participated in any other visits? Can you tell me about them?
8. How have you perceived your relationship with your child health nurse (CHC nurse)?
9. Have you received any support from the child health center (CHC)? Tell me about it:
   1. Parenthood
   2. Fatherhood
   3. Child-related questions
   4. Shared parenting
   5. Questions related to mental health
10. Can you describe how you have experienced these visits?
    1. Were there any benefits to participating?
    2. Did your participation have any consequences?
11. Can you describe how these visits to the child health center (CHC) were experienced by the mother?
12. Is there anything else you would like to say about child health nurses involving and supporting fathers? Can you tell me more?
